# Reducing Annotation Burden Through Multimodal Learning

**DOI:** 10.3389/fdata.2020.00019

**Published:** 2020-06-02

**Authors:** Kevin Lopez, Samah J. Fodeh, Ahmed Allam, Cynthia A. Brandt, Michael Krauthammer

**Affiliations:** ^1^Program of Computational Biology and Bioinformatics, Yale University, New Haven, CT, United States; ^2^Department of Emergency Medicine, Yale School of Medicine, New Haven, CT, United States; ^3^Department of Quantitative Biomedicine, University of Zurich, Zurich, Switzerland; ^4^VA Connecticut Healthcare System, West Haven, CT, United States

**Keywords:** multimodal, deep-learning, CNN, Chest X-Ray, medical imaging

## Abstract

Choosing an optimal data fusion technique is essential when performing machine learning with multimodal data. In this study, we examined deep learning-based multimodal fusion techniques for the combined classification of radiological images and associated text reports. In our analysis, we (1) compared the classification performance of three prototypical multimodal fusion techniques: *Early, Late*, and *Model* fusion, (2) assessed the performance of multimodal compared to unimodal learning; and finally (3) investigated the amount of labeled data needed by multimodal vs. unimodal models to yield comparable classification performance. Our experiments demonstrate the potential of multimodal fusion methods to yield competitive results using less training data (labeled data) than their unimodal counterparts. This was more pronounced using the *Early* and less so using the *Model and Late* fusion approaches. With increasing amount of training data, unimodal models achieved comparable results to multimodal models. Overall, our results suggest the potential of multimodal learning to decrease the need for labeled training data resulting in a lower annotation burden for domain experts.

## Introduction

There is an abundance of multimodal data in the biomedical domain. For example, in electronic health record systems, patients are characterized by multimodal data derived from imaging, genetic, electrophysiological, and many other examinations. Data scientists capitalize on this fact by constructing machine learning models built on two or more data modalities. Examples include the linking of histopathological with proteomic features in the pathology domain (Krishnamurthy et al., 2018; Liu et al., 2018; Yin et al., 2018) or the pairing of text and images modalities derived from biomedical publications (Fodeh et al., 2012, 2013; Andrearczyk and Müller, 2018). In our work, we are interested in both the performance gain and the amount of labeled data needed when comparing unimodal vs. multimodal machine learning approaches. We hypothesize that building machine learning models that exploit the multimodal nature of the data in the biomedical domain offers an advantage in reducing the number of labeled examples required for training such models. Annotating images is an expensive task as it requires fine knowledge about the different types of cancer and consumes extended periods of time from experts. As discussed in Rozenberg et al. (2019) the cost of annotation plagues many different computer vision tasks, which depend on vast amount of training data. This is exaggerated in medical imaging as the labeler is usually a physician, resulting in much higher labeling costs (Rozenberg et al., 2019). Our studies thus investigate the question whether multimodal learning may reduce the cost of annotation by reducing the amount of labeled images needed to reach competitive image classification performance. We constructed multiple experiments to compare three different multimodal fusion strategies (*Early, Late*, and *Model*-based fusion techniques) and investigated how the models perform at varying dataset sizes (varying the number of training examples).

## Materials and Methods

### Data Description

**Figure 1:**
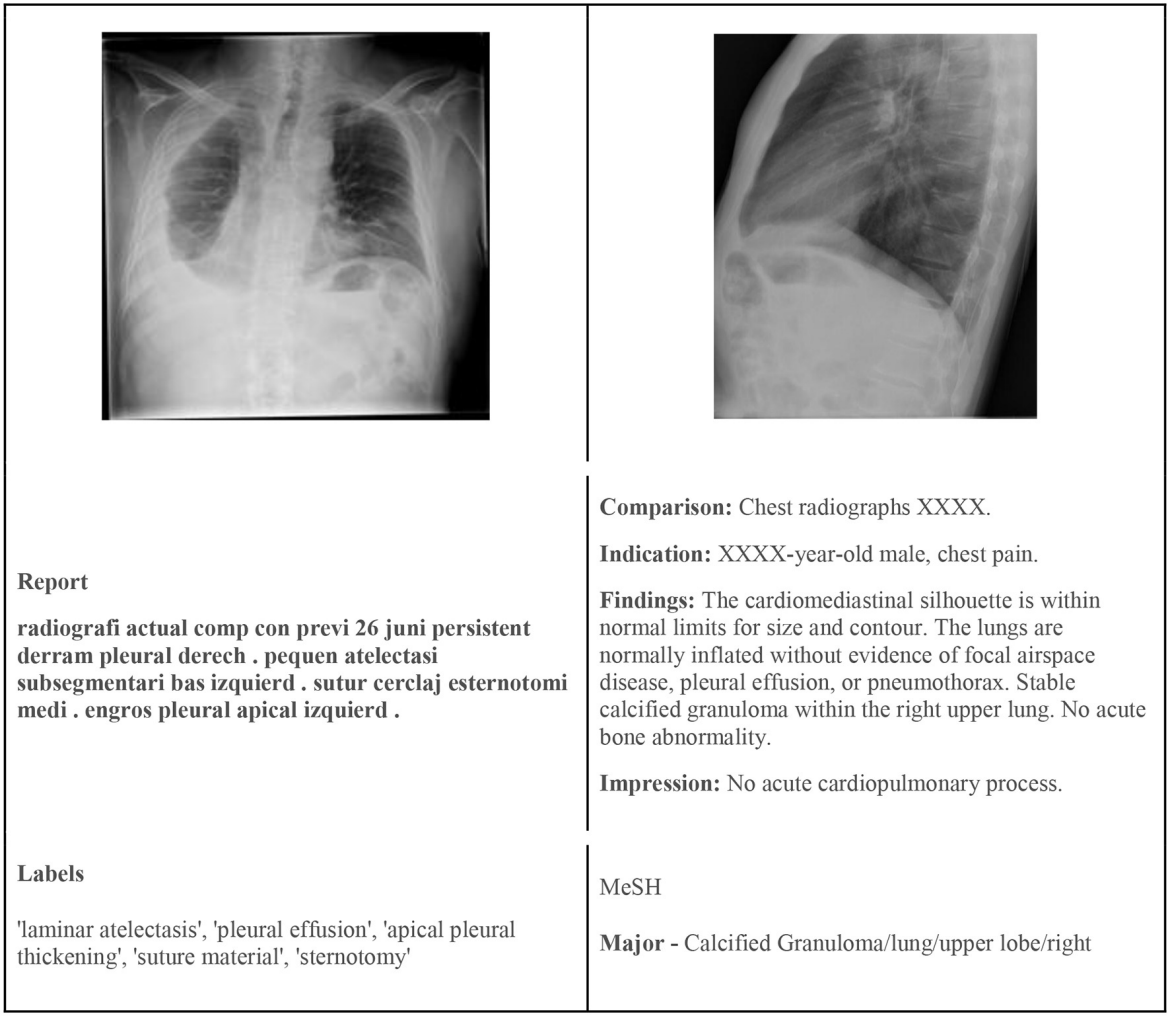
**(Left)** Sample image from PadChest with findings. **(Right)** Sample image from the Indiana Chest X-Ray dataset with MeSH annotation.

To test or models we used two publicly available chest X-ray datasets—the PadChest and Indiana Chest X-Ray datasets. Both datasets have image and text data modalities. Figure 1 shows example images and their corresponding labels and text from the two datasets.

#### PadChest Dataset (Bustos et al., 2019)

Contains over 160,000 images from 67,000 patients. Most samples contain frontal and lateral images with associated radiology reports. All images are of size 224 × 224. The reports are in Spanish and labeled with 174 different radiographic findings and 19 differential diagnoses, 27% of which were labeled by trained physicians. The remaining were labeled by a recurrent neural network. Following earlier work, we simplified image annotations by only using top-level PadChest label categories and restricted experiments to frontal images only. Lateral images have been reported to not provide much benefit and to be difficult to interpret (Gaber et al., 2005; Kluthke et al., 2016; Bertrand et al., 2019). Following the experiments done by Bertrand et al. (2019) we applied class weights to the positive labels to counteract the label imbalance. This left us with a dataset containing 8,192 images with a total of 11 different image labels.

#### Indiana Chest X-Ray Dataset (Demner-Fushman et al., 2016)

Contains 7470 images (frontal and lateral) of 3955 patients with their corresponding MeSH term-annotated radiology reports. As the images in this dataset are of different sizes, we normalized the images by resizing them to 256 × 256 pixels and center cropping them to 224 × 224 pixels. While we made use of all available lateral and frontal images of the Indiana dataset, our model was able to accommodate situations in which a patient had only one of the two images or had more than 2 images. We labeled images as “normal” or “abnormal,” respectively, based on the MeSH terms, resulting in 2,696 “normal” and 4,774 “abnormal” images. We measured a slight label imbalance for abnormal to normal images of 1:1.77.

### Models Framework

We used convolutional neural networks (CNN) (Lecun et al., 1998) and neural word embeddings as the foundation to investigate multimodal learning using *Early, Late*, and *Model*-based fusion techniques.

### Convolutional Neural Networks

A convolutional neural network (CNN) is a feed-forward neural network consisting of multiple layers such as *convolutional, pooling*, and *fully-connected* (FC) layers.

#### Convolutional Layer

Convolutional layer is composed of kernels (i.e., a 2D arrangement of weights in a matrix form) that are convolved with the features of the previous layer (such as the input layer) to produce feature maps. Every entry in the resulting feature map is computed by taking the sum of element-wise multiplication (Hadamard product) of the weights in the kernel and the corresponding input of previous layer where the kernel overlaps. After the convolution operation is done (i.e., convolving the kernel with the input/previous layer), a bias term is added followed by a non-linear operation to obtain a feature map. Multiple kernels are applied, and the resulting feature maps are stacked and processed in the next layers.

#### Pooling Layer

Pooling layer includes kernels/filter but with no trainable weights, that slides over the feature maps (as input) based on a defined horizontal and vertical stride size and computes a summary score such as a maximum or average score for every region of overlap. As a result, in the pooling layer we can change the size of the generated feature maps by specifying the stride and padding values such that the size of the feature maps decreases as we progress into subsequent layers in the network (i.e., equivalent to subsampling).

#### Fully-Connected Layer (FC)

Fully-connected layer (FC) takes an input vector from the reshaped feature maps generated in the last convolutional/pooling layers and applies an affine transformation followed by non-linear element-wise operation.

#### Output Layer

Output layer wherein the computed vector of activations in the penultimate layer is passed to generate a probability distribution over the outcome labels (i.e., the 2 classes in case of the Indiana CXR dataset, and 11 classes for the frontal images in the PadChest dataset).

In our experiments, we implemented CNN using the Keras abstraction of the Tensorflow framework. For both the PadChest and the Indiana CXR datasets, we used the DenseNet121 (Huang et al., 2018) architecture as a base CNN model, which has a mixture of convolutional layers and dense layers that have been shown to be successful in many image processing/classification competitions (i.e., Large Scale Visual Recognition Challenge (ILSVRC2014). It has been shown that the short connections between CNNs layers in the DenseNet121 architecture can be more efficient to train. In addition, DenseNet121 can mitigate the vanishing gradient problem, reduce the parameter count, and strengthen feature propagation (Huang et al., 2018).

### Data Modalities (Image and Text Modalities)

In this section we describe the preprocessing steps on the two data modalities (images and text reports/labels) used in our models.

#### Images

All images were processed to have uniform size of 224 × 224 pixels and one channel. To this end, the Indiana CXR dataset's images we resized, and center cropped to yield an image size of 224 × 224.

#### Text

Words were represented using the one-hot encoding (commonly known by 1-of-K encoding, where K is the size of the vocabulary). To process the text describing the images, we used Word2Vec (Mikolov et al., 2013) to generate neural word embeddings, with embeddings being defined as continuous representations (i.e., vectors with real number components ∈ R^d^ where d is the embedding dimension) (Lebret and Collobert, 2013). Of the two available Word2Vec models (1) continuous bag of words (CBOW) and (2) skip-gram, we used the latter for its better performance on infrequent words. We converted the text associated with images into embeddings using a model that was trained on the text corpus itself. We limited the number of words in the text to 200 words, each represented by a vector of 224 dimensions. The whole report is then represented by a matrix $X_{text}\in R^{200x224}$ (200 tokens, each represented as an embedding vector).

### Fusion Models

We experimented with three types of multimodal fusion methods: (1) Early, (2) Late, and (3) Model fusion. The following sections detail the models' inputs and formulations.

#### Early Fusion

**Figure 2:**
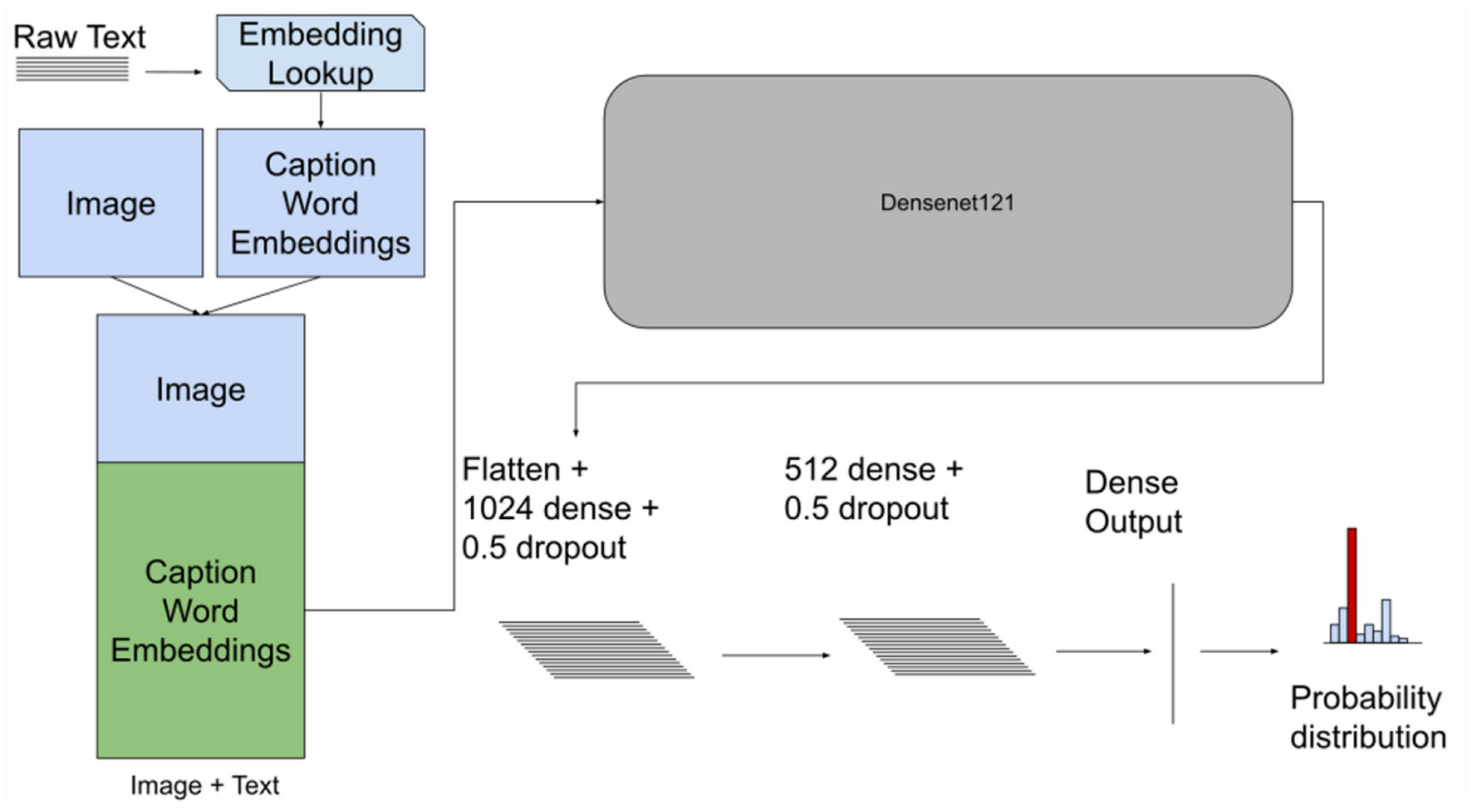
Early fusion model architecture with the concatenated input image and text for both datasets. This model takes images and the embedding matrix for the associated text and concatenates them together. This new matrix is then passed to a CNN as an augmented “image”.

The *Early* fusion method concatenates features from each modality into a single input to the model (Atrey et al., 2010; Baltrušaitis et al., 2019). In this case, we were concatenating the image input with the text input. The concatenation for both datasets was done by stacking the reshaped image matrix $X_{image}\in R^{224x224xC}$ with a text matrix $X_{text}\in R^{200x224xC}$ representing the transformed text from the word embedding model to obtain a final input representation $X_{input}\in R^{424x224xC}$, where *C* is the number of channels. Figure 2 depicts the model architecture with the concatenated input.

A CNN model was used to process the concatenated input. For both the PadChest and Indiana CXR datasets we used a DenseNet121 model and the feature extractor to process the concatenated input. Then two fully-connected (FC) layers were used followed by an output layer with *softmax/sigmoid* activation to predict the probability of each of the outcome classes as shown in Figure 2.

#### Late Fusion

**Figure 3:**
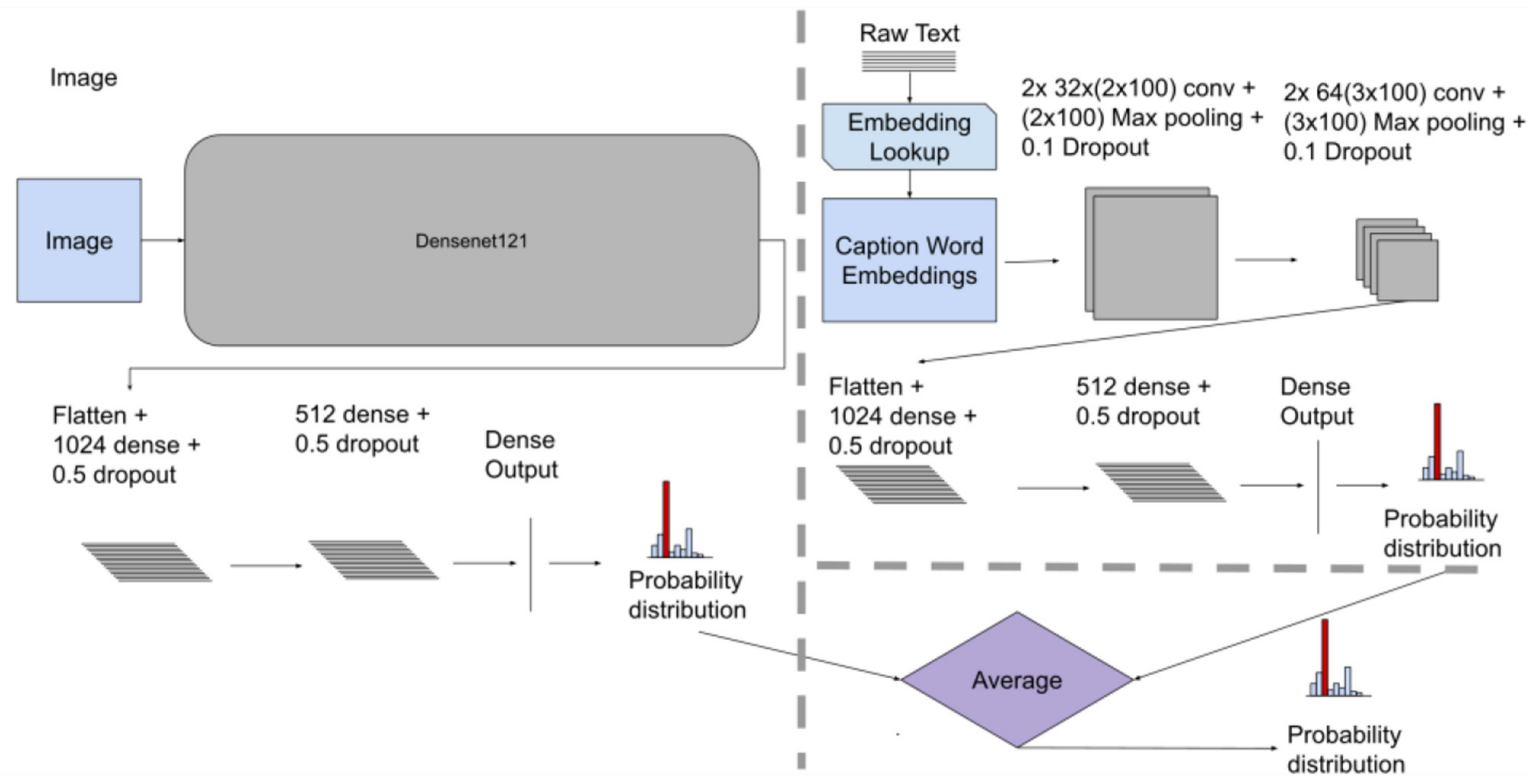
Late fusion model architecture, showing individual unimodal CNN models for images and text along with the averaging fusion mechanism. This method takes independent models and aggregates their results giving a final probability distribution. For both datasets we used the CNN architecture as shown above.

The Late fusion method used two separate models (i.e., two unimodal architectures for processing the images and text separately) with subsequent aggregation of the model decisions using averaging of the results from each architecture. The *Image Only* model had the same architecture as described in the *Early Fusion* section (see Figures 2, 3 for comparison). The *Text Only* model used convolutional layers with “wide” kernels (i.e., rectangular kernels that span the whole embedding dimension such as 2 × *d* and 3 × *d* where *d* is the embedding dimension). Using CNN for processing word embeddings with “wide” kernels has shown competitive results in text classification problems (Kim, 2014). Each of the *Image Only* and *Text Only* trained models generated a probability distribution for the classes (two for Indiana Chest X-Ray, 11 for the PadChest dataset) representing their outcome decision. The fusion mechanism was done by aggregating the outcome decision of each model (i.e., averaging both distributions) to obtain a final decision (Atrey et al., 2010; Baltrušaitis et al., 2019). Figure 3 displays the architecture of the Late fusion model.

#### Model Fusion

The Model fusion introduces a latent layer to combine the image and text data modalities. It combines outputs of the last convolutional layers from each model. The combined vector from the introduced latent layer is subsequently fed as an input to further layers (i.e., two FC layers and an output layer—*softmax* or *sigmoid*), resulting in a single probability distribution. This approach represents an “end-to-end” fusion model that is trained using both modalities where the fusion mechanism is part of the model training process.

**Figure 4:**
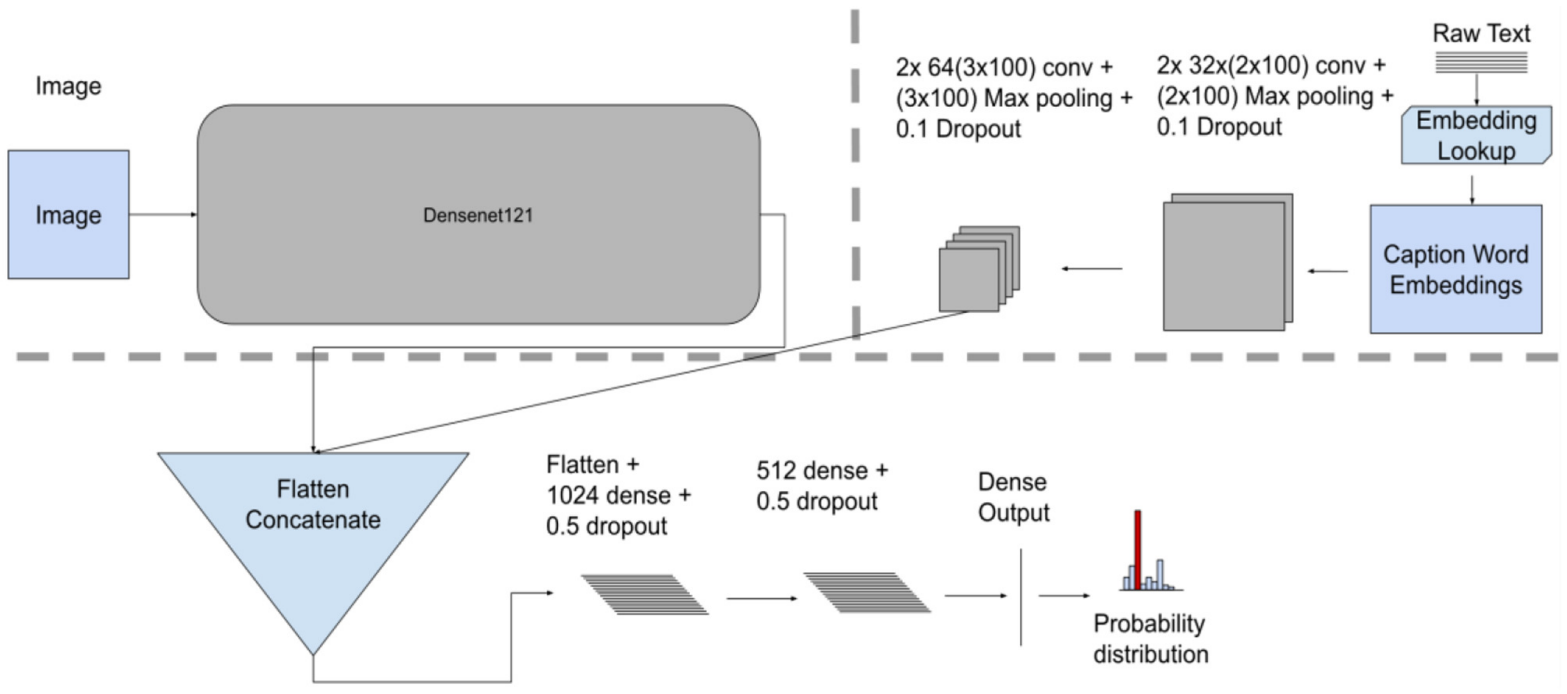
Model fusion model architecture, showing individual unimodal CNN feature extractors for images and text along with the concatenation fusion mechanism, a terminal network consisting of three dense layers.

Unlike the Late fusion model, this approach takes the generated feature maps in the last convolutional layers from both modalities and concatenates them as one input to the fully connected layer (see Figure 4 for model architecture).

### Experiments

#### Comparing Performances of Unimodal and Multimodal Models by Varying Size of Labeled Data

**Figure 5:**
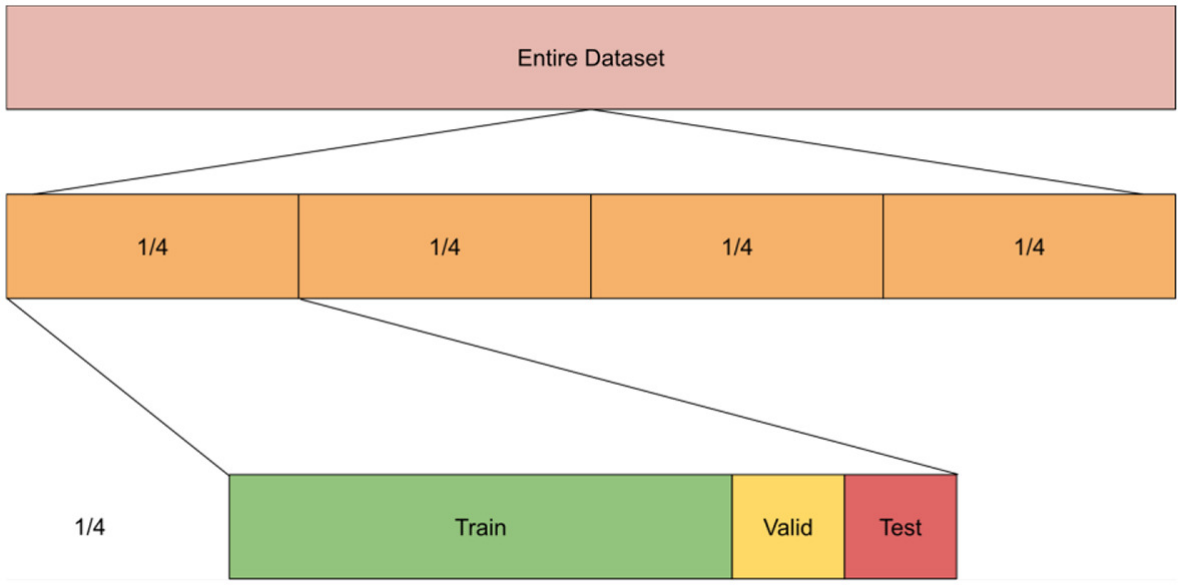
Experiment setup showing 4 splits wherein each split we divide it into training, valid, and testing sets.

We compared performance of unimodal and multimodal models at different values of *n* where n is the number of splits of labeled data. The rational of this experiment is to check whether we can reduce the burden of manual annotation by using multimodal models. Specifically, we compare the performance of unimodal models trained using all labeled data (*n* = 1) to multimodal models when trained on half the data (*n* = 2) or one-fourth of the data (*n* = 4). That is, we partitioned the data into *n* chunks where *n* varies for every experiment such that *n*∈4, 2, 1. For example, when *n* = 1, the whole dataset was used in the experiment, while *n* = 4 means we had 4 partitions of the dataset, each was used separately in the training/testing of the models. Within each partition, we split the data into 80% training, 10% validation, and 10% testing subsets. We repeated all experiments 5 times and averaged the results. We used the average performance of each of the partitions/chunks to get the scores across all the partitions (see Figure 5 for illustration). Additionally, given that both datasets had multiple images per patient, we took measures to ensure that each sample (i.e., all data from a patient) is either in the training or valid or testing sets. We report AUC (area under curve), precision, recall and the F1 score. To calculate the latter, we used the following formula: $F1=2\times \frac{Precision\;\times \;Recall}{Precision\;+\;Recall}$. All models were trained for 80 epochs with a batch size of 8 using the “ADAM” optimizer.

We use the **ModelName(text kernel, image kernel, pretraining)** convention for describing the models used in the paper, where

- **ModelName:** corresponds to the type of model used (i.e., unimodal vs. multimodal along with the fusion method used),
- **Text kernel:** refers to the type of kernel used for the text channel (Square vs. Wide).
- **Image kernel:** refers to the type of kernel used for the image channel (i.e., Square vs. Wide) and
- **Pretrained:** signifies whether pretraining was performed or not (i.e., using unimodal pretrained weights as initialization to the multimodal weight matrices).

#### Comparing Performance of Unimodal and Multimodal Models

To investigate the added value of our three multimodal models, we compared their performance to unimodal models. In this experiment, we used the same number of labeled training data (*n* = 1). We used two unimodal models to conduct our experiments: *Image Only (Square)* and *Text Only (Wide)*. The *Image Only (Square)* model is a CNN trained on images using a “square” kernel to extract image features. The *Text Only (Wide)* model is a CNN with a “wide” kernel trained on text features.

#### Models' Setup and Parameters

All models used the same hyperparameters and splits into training/validation and testing data. Batch normalization layers were part of the models' architecture and *ReLU* (Krizhevsky et al., 2012) was used as an activation function for the *hidden* layers. We used a scheduled learning rate with “ADAM” as an optimizer (Kingma and Ba, 2014). As a loss function, we used *categorical cross-entropy* for the Indiana CXR dataset and *binary cross entropy* for the PadChest dataset. Model regularization *dropouts* (Srivastava et al., 2014) were set to prob. = 0.5 in the fully connected layers. In the case of the PadChest dataset, we used the *sigmoid* activation for the *output* layer and *softmax* for the Indiana dataset. While training, we augmented the input images using horizontal flips (prob. = 0.5) and converting them to grayscale for the Indiana CXR dataset to stabilize training.

For the PadChest dataset, we followed a similar setup to Bertrand et al. (2019) using only frontal images and trained on DenseNet121 (Huang et al., 2018). However, instead of having a Global Average Pooling (Lin et al., 2014) layer followed by a fully connected layer, we flattened the feature maps and appended two fully connected layers (ReLU activation) followed by a batch normalization layer, a dropout layer (prob. = 0.5) and finally a fully connected layer as output.

## Results

### Comparing Performances of Unimodal and Multimodal Models to Reduce Annotation Burden

Table 1 shows the AUC, F1, precision, and recall scores for the *PadChest* dataset. All measures are reported at varying sizes of the training set: *n* ε {1,2,4}. For each n, we ran the model 5 times and calculated an average performance. We find that for or *n* > 1 multimodal fusion approaches exhibited less performance degradation compared to unimodal models. At *n* = 2, *Early Fusion (Square)* model scored 0.96 AUC which is comparable to its performance at *n* = 1 (0.98 AUC)—a degradation of 2%. Also, at ¼ of the dataset, the AUC dropped by only 5.1% (0.98 vs. 0.93 AUC, Table 1). For unimodal models, which were outperformed by multimodal models for all values of n, the performance degradation was worse: at *n* = 4 (¼ of the dataset), the best performing unimodal model *Text Only (Wide)* exhibited a performance degradation of 10.4% (0.96 vs. 0.86 AUC).

**Table 1:** Average F1, precision, recall, and AUC scores of every model for each *n* performed 5 times and averaged together for the PadChest dataset.

**Model**	***N*** **=** **1**				***N*** **=** **2**				***N*** **=** **4**			
	**F1**	**Precision**	**Recall**	**AUC**	**F1**	**Precision**	**Recall**	**AUC**	**F1**	**Precision**	**Recall**	**AUC**
Image Only (Square)	0.28	0.40	0.22	0.72	0.24	0.40	0.18	0.71	0.21	0.34	0.15	0.69
Text Only (Wide)	0.70	0.80	0.62	0.96	0.52	0.69	0.42	0.92	0.34	0.51	0.26	0.86
Early Fusion (Square)	0.84	0.89	0.79	0.98	0.75	0.83	0.67	0.96	0.62	0.75	0.53	0.93
Early Fusion (Wide)	0.63	0.77	0.53	0.95	0.43	0.67	0.32	0.90	0.25	0.40	0.19	0.83
Late Fusion (Wide, Square)	0.64	0.86	0.51	0.95	0.46	0.72	0.34	0.90	0.32	0.44	0.25	0.80
Late Fusion (Wide, Square, Pretrained)	0.71	0.91	0.58	0.96	0.55	0.78	0.42	0.92	0.37	0.53	0.28	0.84
Model Fusion (Wide, Square)	0.70	0.80	0.63	0.95	0.56	0.71	0.46	0.92	0.38	0.56	0.28	0.85
Model Fusion (Wide, Square, Pretrained)	0.79	0.85	0.74	0.96	0.64	0.75	0.55	0.93	0.43	0.61	0.34	0.87

Similar experiments were performed on the Indiana dataset. All unimodal and multimodal models were run 5 times for each n and the average AUC, F1, precision, and recall scores calculated. We observed similar results (Table 2) as above, with *Early Fusion (Wide)* achieving a 0.93 AUC value at *n* = 1, compared to an AUC = 0.90 at *n* = 2, a drop of 3.2%. While unimodal models showed competitive performances, multimodal models still resulted in higher AUCs values across all *n*s.

**Table 2:** Average F1, precision, recall, and AUC scores of every model for each *n* performed 5 times and averaged together for the Indiana Chest X-Ray dataset.

**Model**	***N*** **=** **1**				***N*** **=** **2**				***N*** **=** **4**			
	**F1**	**Precision**	**Recall**	**AUC**	**F1**	**Precision**	**Recall**	**AUC**	**F1**	**Precision**	**Recall**	**AUC**
Image Only (Square)	0.46	0.52	0.42	0.61	0.38	0.53	0.30	0.57	0.38	0.49	0.30	0.56
Text Only (Wide)	0.88	0.80	0.97	0.92	0.86	0.77	0.97	0.90	0.79	0.72	0.87	0.84
Early Fusion (Square)	0.89	0.87	0.90	0.92	0.86	0.85	0.87	0.89	0.82	0.80	0.85	0.86
Early Fusion (Wide)	0.91	0.90	0.92	0.93	0.86	0.79	0.94	0.90	0.79	0.69	0.91	0.84
Late Fusion (Wide, Square)	0.89	0.82	0.96	0.93	0.84	0.75	0.95	0.88	0.79	0.7	0.89	0.84
Late Fusion (Wide, Square, Pretrained)	0.89	0.83	0.96	0.93	0.86	0.78	0.96	0.90	0.82	0.74	0.91	0.86
Model Fusion (Wide, Square)	0.86	0.76	0.97	0.91	0.84	0.74	0.97	0.89	0.80	0.69	0.95	0.85
Model Fusion (Wide, Square, Pretrained)	0.86	0.78	0.97	0.91	0.85	0.76	0.97	0.89	0.81	0.71	0.94	0.86

### Comparing Performance of Unimodal and Multimodal Models

For the *PadChest* dataset, *Early Fusion (Square)* showed the highest performance across all *n*s, besting the highest performing unimodal model *Text Only (Wide)* (Table 1). For the multimodal models, *Early Fusion (Square)* yielded an average AUC of 0.98, while *Model Fusion (Wide, Square, Pretrained)* scored 0.96. *Late* and *Model fusion* without pretraining resulted in a slightly lower performance, with *Model Fusion (Wide, Square, randomly initialized)* achieving an AUC of 0.95 at *n* = 1, for example. Looking at the unimodal models in Table 1, the *Text Only (Wide)* model outperformed the *Image Only (Square)* model at every value of *n*. At *n* = 1, the *Text Only (Wide)* model scored an average AUC of 0.96 while the *Image Only (Square)* scored an AUC of 0.72.

For the Indiana dataset (Table 2), the multimodal models *Early Fusion (Wide)* and *Late Fusion (Wide, Square)* are among the best performing models with an AUC of 0.93. Unlike the results above, pretraining did not boost the performance of fusion approaches. The unimodal model *Text Only (Wide)* outperformed the *Image Only (Square)* model at every value of *n*, with *Text Only (Wide)* scoring an average AUC of 0.92 and the *Image Only (Square)* an average AUC of 0.61 at *n* = 1.

**Figure 6:**
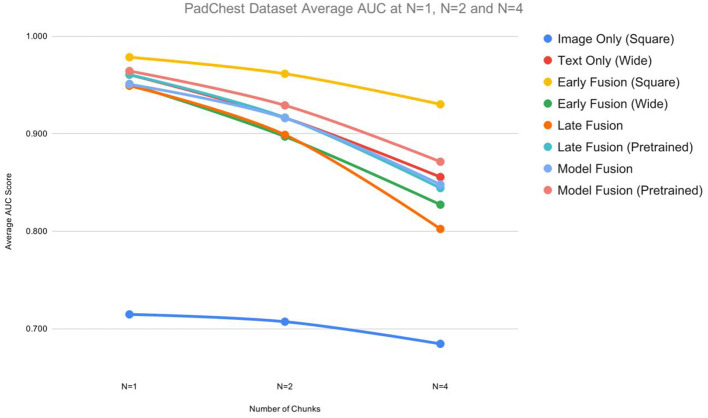
Averaged AUC scores for the PadChest dataset for n ε {1,2,4}.

**Figure 7:**
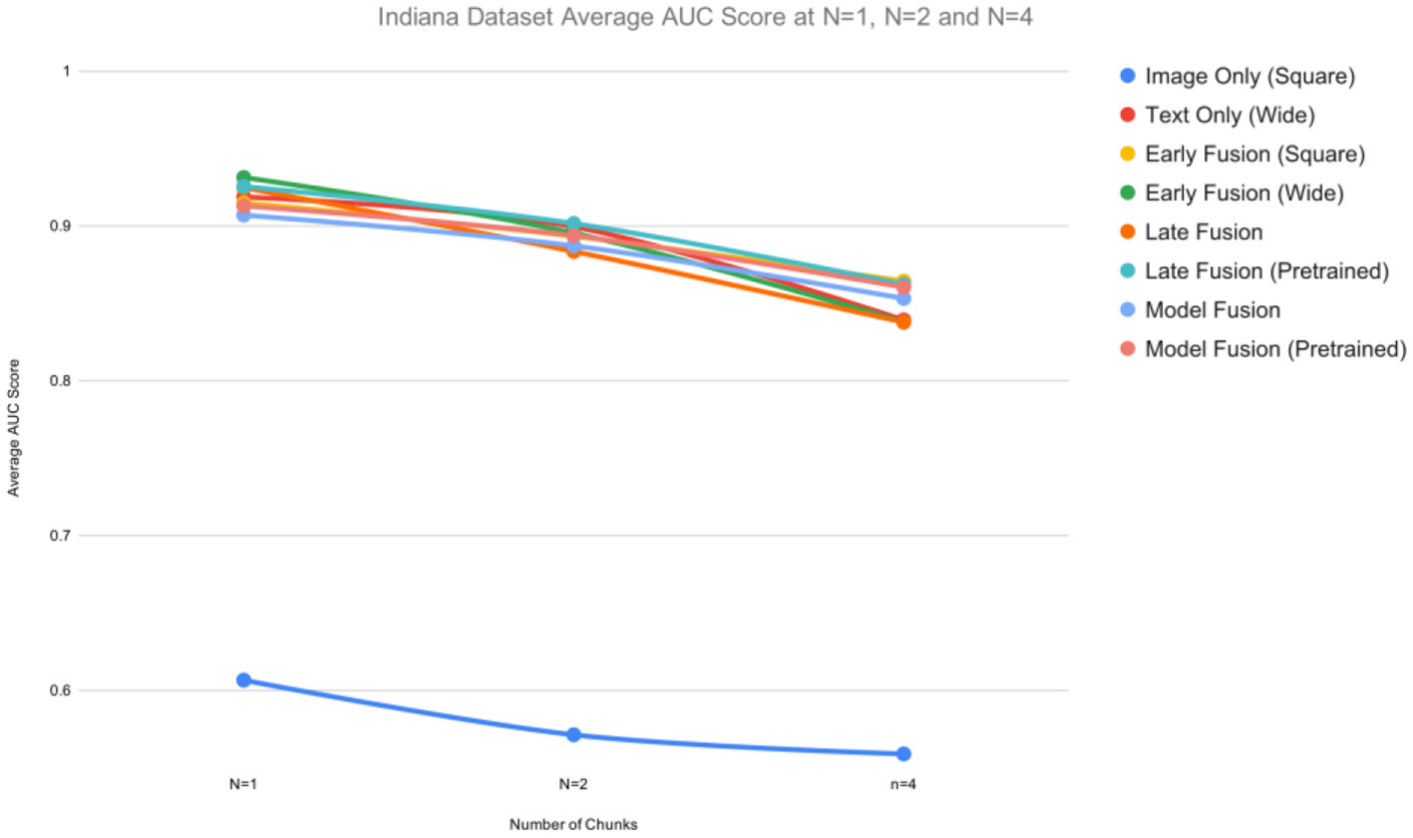
Averaged AUC scores for the Indiana CXR dataset for n ε {1,2,4}.

Figures 6, 7 provide a graphical summary of the AUC performances across the models.

## Discussion and Conclusion

Our experiments show that multimodal learning provides advantages over unimodal learning when performing classification of radiological images and their associated text reports. With regard to the fusion technique, *Early Fusion* achieved the highest performance across two independent biomedical datasets. We suspect *Early Fusion (Square)* did well on the PadChest dataset due to DenseNet121s' ability to propagate features deep into the network. On the Indiana dataset, we believe *Early Fusion (Wide)* performed the best because annotators used the text to assign the labels. Interestingly, we find that—compared to their unimodal counterparts—multimodal models have the potential to reduce the annotation burden by reducing the number of training samples required to train such models. For example, for the PadChest dataset, performance decrease was half that of unimodal models when training on a dataset comprised of ¼ of the total data (a decrease of 5.1 vs. 10.4%). This is an important finding that can serve toward reducing annotation burden, which usually comes with high cost in terms of time and human labor. Instead of annotating the entire dataset for building the classification model, a smaller annotated dataset can be used to deliver competitive classification performance. Other advantages are the potential to shorten training time, reduce the cost of annotation, and lessen fatigue and pressure exerted on annotators (Egleston et al., 2011; Amgad et al., 2019). These advantages may reduce the probability of having miss-annotations or low-quality annotated datasets.

In general, *Early Fusion* did the best of the three fusion methods. We suspect the square variant's performance is due to DenseNet121's ability to strengthen both feature propagation and the reuse of features, allowing the model to push features deeper into the network. We also observed that—for the Indiana, and less so for the PadChest dataset- the text modality (i.e., radiology reports) had a large influence on the performance of the trained models, as reflected by an AUC of 0.92 of the text-only compared to an AUC of 0.61 of the image-only model at *n* = 1. We believe this result may reflect the process of annotating the datasets. In the Indiana CXR case, annotators focused on the text to assign the labels for the reports (Demner-Fushman et al., 2016), with the results that discriminative features are mostly found in the text modality of the dataset. Finally, although wide kernels are known to work well when classifying text (Kim, 2014), our results did not give a clear picture when comparing square vs. wide kernels for the different fusion approaches.

In the future, we plan to investigate several avenues. One is the use of additional modalities, such as incorporating genomic and clinical data, or apply our models to other types of medical images, such as CTs or MRIs. We also plan to investigate different neural architectures and models, such as recurrent neural networks and transformer networks (Vaswani et al., 2017). Moreover, we would like to incorporate “attention-mechanisms” (Vaswani et al., 2017) to identify the feature contributions across the modalities in addition to investigating semi-supervised learning with multimodal learning.

In conclusion, we evaluated different fusion architectures for classifying radiology exams from two data sets using both image and text modalities. We found that fusion-based models achieved slightly better performance compared to models using a single modality and that they show robust performance in experiments with reduced training set sizes. We conclude that multimodal learning leads to competitive performance in classifying radiology exams and may help to reduce annotation burden on domain experts.

## Data Availability Statement

The datasets analyzed for this study can be found in the Open Access Biomedical Image Search Engine https://openi.nlm.nih.gov/imgs/collections/NLMCXR_png.tgz and at the Medical Imaging Databank of the Valencia Region http://bimcv.cipf.es/bimcv-projects/padchest/.

## Author Contributions

KL developed experiments and models, conducted experiments, wrote initial draft, and edited drafts. SF designed experiments and edited drafts. AA, CB, and MK edited draft.

## Conflict of Interest

The authors declare that the research was conducted in the absence of any commercial or financial relationships that could be construed as a potential conflict of interest.

## References

[B1] Amgad M. Elfandy H. Hussein H. Atteya L. A. Elsebaie M. A. T. Abo Elnasr L. S.. (2019). Structured crowdsourcing enables convolutional segmentation of histology images. Bioinformatics 35, 3461–3467. 10.1093/bioinformatics/btz083 30726865 PMC6748796

[B2] Andrearczyk V. Müller H. (2018). “Deep multimodal classification of image types in biomedical journal figures,” in Experimental IR Meets Multilinguality, Multimodality, and Interaction. Lecture Notes in Computer Science, eds P. Bellot, C. Trabelsi, J. Mothe, F. Murtagh, J. Y. Nie, L. Soulier, E. SanJuan, L. Cappellato, and N. Ferro (Cham: Springer International Publishing), 3–14. 10.1007/978-3-319-98932-7_1

[B3] Atrey P. K. Hossain M. A. El Saddik A. Kankanhalli M. S. (2010). Multimodal fusion for multimedia analysis: a survey. Multimedia Syst. 16, 345–379. 10.1007/s00530-010-0182-0

[B4] Baltrušaitis T. Ahuja C. Morency L.-P. (2019). Multimodal machine learning: a survey and taxonomy. IEEE Trans. Pattern Anal. Mach. Intell. 41, 423–443. 10.1109/TPAMI.2018.2798607 29994351

[B5] Bertrand H. Hashir M. Cohen J. P. (2019). Do lateral views help automated chest x-ray predictions? arXiv [Preprint]. arXiv:1904.08534. Available online at: http://arxiv.org/abs/1904.08534 (accessed May 11, 2020).

[B6] Bustos A. Pertusa A. Salinas J.-M. Iglesia-Vayá M. (2019). PadChest: A Large Chest X-Ray Image Dataset With Multi-Label Annotated Report. Alicante: University of Alicante 10.1016/j.media.2020.101797 32877839

[B7] Demner-Fushman D. Kohli M. D. Rosenman M. B. Shooshan S. E. Rodriguez L. Antani S. K.. (2016). Preparing a collection of radiology examinations for distribution and retrieval. J. Am. Med. Informatics Assoc. 23, 304–310. 10.1093/jamia/ocv080 26133894 PMC5009925

[B8] Egleston B. L. Miller S. M. Meropol N. J. (2011). The impact of misclassification due to survey response fatigue on estimation and identifiability of treatment effects. Stat. Med. 30, 3560–3572. 10.1002/sim.4377 21953305 PMC3552436

[B9] Fodeh S. J. Brandt C. Luong T. B. Haddad A. Schultz M. Murphy T.. (2013). Complementary ensemble clustering of biomedical data. J. Biomed. Informatics 46, 436–443. 10.1016/j.jbi.2013.02.001 23454721 PMC4007219

[B10] Fodeh S. J. Haddad A. Brandt C. Schultz M. Krauthammer M. (2012). Enhancing clustering by exploiting complementary data modalities in the medical domain. IFIP Adv. Information Commun. Technol. 381, 357–367. 10.1007/978-3-642-33409-2_37 29479376 PMC5823526

[B11] Gaber K. A. Mcgavin C. R. Wells I. P. (2005). Lateral chest X-ray for physicians. J. R. Soc. Med. 98, 310–312. 10.1177/014107680509800705 15994591 PMC1168914

[B12] Huang G. Liu Z. van der Maaten L. Weinberger K. Q. (2018). Densely connected convolutional networks. arXiv [Preprint]. arXiv:1608.06993. Available online at: http://arxiv.org/abs/1608.06993 (accessed May 11, 2020).

[B13] Kim Y. (2014). Convolutional neural networks for sentence classification. arXiv [Preprint]. arXiv:1408.5882. 10.3115/v1/D14-1181

[B14] Kingma D. P. Ba J. (2014). Adam: a method for stochastic optimization. arXiv [Preprint]. arXiv:1412.6980.

[B15] Kluthke R. A. Kickuth R. Bansmann P. M. Tüshaus C. Adams S. Liermann D.. (2016). The additional value of the lateral chest radiograph for the detection of small pulmonary nodules-a ROC analysis. Br. J. Radiol. 89:20160394. 10.1259/bjr.20160394 27605206 PMC5124842

[B16] Krishnamurthy G. Majumder N. Poria S. Cambria E. (2018). A deep learning approach for multimodal deception detection. arXiv [Preprint]. arXiv:1803.00344. Available online at: http://arxiv.org/abs/1803.00344 (accessed May 11, 2020).

[B17] Krizhevsky A. Sutskever I. Hinton G. E. (2012). “ImageNet classification with deep convolutional neural networks,” in Advances in Neural Information Processing Systems, Vol. 25, eds F. Pereira, C. J. C. Burges, L. Bottou, and K. Q. Weinberger (Red Hook, NY: Curran Associates, Inc.), 1097–1105. Available online at: http://papers.nips.cc/paper/4824-imagenet-classification-with-deep-convolutional-neural-networks.pdf (accessed May 11, 2020).

[B18] Lebret R. Collobert R. (2013). Word Emdeddings Through Hellinger PCA.

[B19] Lecun Y. Bottou L. Bengio Y. Haffner P. (1998). Gradient-based learning applied to document recognition. Proc. IEEE 86, 2278–2324. 10.1109/5.726791

[B20] Lin M. Chen Q. Yan S. (2014). Network in network. arXiv [Preprint]. arXiv:1312.4400. Available online at: http://arxiv.org/abs/1312.4400 (accessed May 11, 2020).

[B21] Liu K. Li Y. Xu N. Natarajan P. (2018). Learn to combine modalities in multimodal deep learning. arXiv [Preprint]. arXiv:1805.11730. Available online at: http://arxiv.org/abs/1805.11730 (accessed May 11, 2020).

[B22] Mikolov T. Chen K. Corrado G. Dean J. (2013). Efficient estimation of word representations in vector space. arXiv [Preprint]. arXiv:1301.3781. Available online at: http://arxiv.org/abs/1301.3781 (accessed May 11, 2020).

[B23] Rozenberg E. Freedman D. Bronstein A. (2019). Localization with limited annotation for chest x-rays. arXiv [Preprint]. arXiv:1909.08842. Available onine at: http://arxiv.org/abs/1909.08842 (accessed May 11, 2020).

[B24] Srivastava N. Hinton G. Krizhevsky A. Sutskever I. Salakhutdinov R. (2014). Dropout: a simple way to prevent neural networks from overfitting. J. Mach. Learn. Res. 15, 1929–1958.

[B25] Vaswani A. Shazeer N. Parmar N. Uszkoreit J. Jones L. Gomez A. N.. (2017). “Attention is all you need,” in Advances in Neural Information Processing Systems 30, eds I. Guyon, U. V. Luxburg, S. Bengio, H. Wallach, R. Fergus, S. Vishwanathan, and R. Garnett (Curran Associates, Inc.), 5998–6008. Available online at: http://papers.nips.cc/paper/7181-attention-is-all-you-need.pdf (accessed May 11, 2020).

[B26] Yin Y. Shah R. R. Zimmermann R. (2018). “Learning and fusing multimodal deep features for acoustic scene categorization,” in Proceedings of the 26th ACM International Conference on Multimedia MM '18 (Seoul: Association for Computing Machinery), 1892–1900. 10.1145/3240508.3240631

